# From Methane to Nanodiamond Precursors in Water: Superacid‐like Condensation Pathways Under Extreme Conditions

**DOI:** 10.1002/anie.202520364

**Published:** 2025-11-26

**Authors:** Thomas Thévenet, Axel Dian, Matteo Cioni, Alexis Markovits, Sandro Scandolo, Arthur France‐Lanord, Flavio Siro Brigiano

**Affiliations:** ^1^ Laboratoire de Chimie Théorique Sorbonne Université CNRS UMR 7616, 4 place Jussieu Paris 75005 France; ^2^ Muséum National d'Histoire Naturelle, UMR CNRS 7590 Institut de Minéralogie, de Physique des Matériaux et de Cosmochimie, IMPMC Sorbonne Université Paris F‐75005 France; ^3^ The Abdus Salam International Centre for Theoretical Physics Str. Costiera, 11 Trieste 34151 Italy

**Keywords:** Ab initio molecular dynamics, CH_5_^+^ formation in water, Hydrocarbon chemistry under extreme conditions, Machine learning potentials, Superacid chemistry

## Abstract

The chemical behavior of water and hydrocarbons under extreme pressures and temperatures lies at the heart of processes shaping planetary interiors, influences the deep carbon cycle, and underpins innovative high‐temperature, high‐pressure material synthesis. Recent experiments have shown that simple hydrocarbons immersed in water under extreme conditions transform into heavier hydrocarbons and nanodiamonds. However, the chemistry of water in these regimes, and its role in driving hydrocarbon condensation, remain poorly understood. Here, using atomistic simulations techniques, we show that water under extreme conditions acts like a strong superacid, protonating hydrocarbons and forming transient pentacoordinated carbocations such as CH_5_^+^. These fleeting species can either transfer the proton to neighboring water species, or release molecular hydrogen to generate highly reactive carbocations that drive hydrocarbon chain growth. These mechanisms parallel the superacid catalyzed hydrocarbon condensation at ambient conditions that was discovered in the work of George Olah, who demonstrated that methane polycondensation proceeds via transient pentacoordinated ions in superacids. Our work shows that the same non‐classical carbocation chemistry emerges in water under extreme conditions, leading to nanodiamond precursors. These findings reveal the existence of superacid‐like hydrocarbon condensation in water, and provide a unifying reaction network that explains chemical transformations in environments such as planetary interiors.

## Graphical abstract

Under extreme pressure and temperature, superionic water acts as a strong superacid, catalyzing methane polycondensation through pentacoordinated carbonium ions (CH_5_^+^). This discovery reveals the non‐classical carbocation chemistry underlying the early stages of diamond formation in water under extreme conditions.

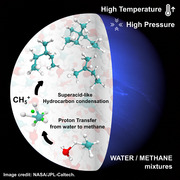

## Introduction

The chemistry of carbon based compounds in water under extreme conditions is of critical importance across several fields, ranging from planetary science,^[^ ^1^, ^2^, ^3^, ^4^, ^5^, ^6^ ^]^ terrestrial geochemistry^[^ ^7^, ^8^, ^9^ ^]^ to the origin of life,^[^ ^9^, ^10^ ^]^ as well as for the high‐pressure and high‐temperature (HPHT) synthesis of new materials.^[^ ^11^, ^12^, ^13^ ^]^ Within the deep interiors of planets, such as Earth's mantle or the fluid layers of icy giants and sub‐Neptune exoplanets, water, and carbon based compounds are subjected to high pressures and temperatures, driving unique chemical transformations. As pressure and temperature rise, water undergoes increased ionization^[^ ^14^, ^15^, ^16^, ^17^ ^]^ (e.g. 15% of dissociated water^[^ ^18^ ^]^ at 2000 K and 1.8 *g*/*cm* ^3^), creating a highly reactive environment that profoundly impacts the chemical stability and reactivity of dissolved compounds.^[^ ^9^, ^11^, ^19^, ^20^, ^21^ ^]^

One of the most enigmatic transformations under extreme conditions in water is the polycondensation of simple hydrocarbons into heavier hydrocarbons and nanodiamonds. The HPHT synthesis of heavy hydrocarbon mixtures and diamonds carries significant implications for planetary science,^[^ ^1^, ^2^, ^3^, ^4^, ^22^, ^23^, ^24^, ^25^, ^26^, ^27^, ^28^, ^29^, ^30^ ^]^ geochemistry,^[^ ^8^, ^20^, ^31^ ^]^ detonation experiments,^[^ ^11^ ^]^ and petrochemistry.^[^ ^32^ ^]^ In planetary science, the “diamonds in the sky” hypothesis^[^ ^1^ ^]^ posits that, within the interiors of icy giants, methane, and water, the primary constituents of their fluid mantles, transform into diamonds and ionized water, potentially explaining the anomalous magnetic fields and luminosity of these planets. Recent experimental evidence from laser‐heated diamond anvil cell (LHDAC)^[^ ^2^ ^]^ and shock compression experiments^[^ ^3^ ^]^ support this hypothesis by probing the transition of C/H/O mixtures into heavy hydrocarbons, nanodiamonds, and ionized water. Despite these observations, the mechanisms governing hydrocarbon condensation in ionized water remain largely unexplored due to the challenges of characterizing these reactions in situ. Computational methodologies based on quantum chemical calculations, including ab initio molecular dynamics (AIMD),^[^ ^4^, ^5^, ^6^, ^23^, ^25^, ^33^, ^34^, ^35^ ^]^ crystal structure prediction algorithms,^[^ ^36^, ^37^, ^38^, ^39^, ^40^ ^]^ and quantum topological analyses,^[^ ^41^, ^42^, ^43^, ^44^ ^]^ have emerged as powerful tools for gaining molecular‐level insight into the transformations of matter under extreme conditions. In the context of the “diamonds in the sky” hypothesis, computational studies have provided valuable insight into methane reactivity at high pressure and temperature. In their pioneering study, Ancillotto et al.^[^ ^23^ ^]^ used AIMD to study the transformation of pure liquid methane, finding that it begins to dissociate at 4000 K and 100 GPa, forming heavier hydrocarbons and molecular hydrogen. More recently, Lee et al.^[^ ^4^ ^]^ and Militzer^[^ ^5^ ^]^ performed ab initio simulations on H_2_O/CH_4_ and H_2_O/CH_4_/NH_3_ mixtures, systems more representative of the fluid mantles of icy giants. Both studies report the formation of C─C and C─O bonds, promoted by increasing pressure and temperature. Notably, Lee et al.^[^ ^4^ ^]^ found that methane begins to dissociate in water at 3000 K and 50 GPa, significantly milder conditions than those predicted for pure liquid methane.^[^ ^23^ ^]^ Despite significant advances, the fundamental chemistry of hydrocarbon transformation in water under extreme conditions remains largely unknown. Relatedly, recent ab initio simulations by Seeyangnok et al.^[^ ^35^ ^]^ reported non‐classical hydrocarbon chemistry in metallic hydrogen at 500 GPa, characterized by stable six‐coordinated carbon species, illustrating how extreme conditions can profoundly alter the chemistry of organic compounds.

In this study, we investigate the chemical behavior of water/methane mixtures under extreme conditions by simulating their reactivity in pressure–temperature regimes where heavy hydrocarbon and diamond formation have been experimentally observed.^[^ ^2^, ^3^ ^]^ First, using a combination of DFT‐MD and machine‐learning interatomic potentials^[^ ^45^, ^46^ ^]^ (MLIPs), we explore methane condensation pathways in water. Our simulations reveal that water, in such extreme conditions, is capable of protonating methane leading to the formation of non‐classical CH_5_^+^ ions, the same chemical behavior observed for methane in strong superacids.^[^ ^47^, ^48^, ^49^ ^]^ We show that water catalyzes methane polycondensation via these transient pentacoordinated carbonium ions (e.g., CH_5_^+^), a chemistry that closely mirrors the superacid catalyzed condensation of hydrocarbons at moderate temperatures (60–150 °C) and ambient pressure, as described in the studies of Olah et al.^[^ ^47^, ^48^, ^49^ ^]^ Then, using enhanced sampling techniques, we investigate chain elongation, hydrocarbon branching, and quaternary carbon structure formation, which eventually allows us to sketch a mechanistic framework for understanding how high pressure and water ionization drive such transformations. We find that three water acid‐catalyzed and one base‐catalyzed mechanisms underpin the whole reaction network.

## Results and Discussion

### CH_5_^+^ Formation in Water

**Figure 1:**
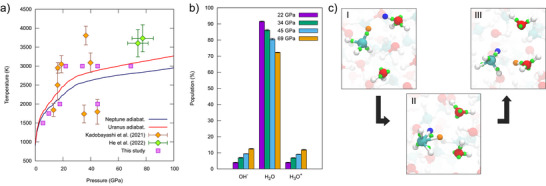
Panel a: Pressure and temperature conditions explored in this study (purple squares), alongside previous LH‐DAC^[^ ^2^ ^]^ (orange rhombuses) and shock wave experiments^[^ ^3^ ^]^ (green rhombuses) on C/H/O mixtures. The solid blue and red lines depict the predicted isentropes of Uranus and Neptune from ref. [51]; Panel b: Population of H_2_O, H_3_O^+^ and OH^−^ species in the 22–69 GPa range at 3000 K. Error bars correspond to a 95% confidence interval. Panel c: Reaction structures with Wannier centers associated to the Grotthuss‐like proton‐hopping mechanism between hydronium ion and methane. The proton donated by water is shown in orange, while the proton donated by methane is shown in blue.

We simulated H_2_O/CH_4_ mixtures using simulation boxes composed of 76 water molecules and 52 methane (488 atoms), a stoichiometry approximately close to the protosolar ratio of heavy nuclei.^[^ ^50^ ^]^ Eight P─T conditions were investigated (purple squares in Figure 1a) that closely approach the adiabats of Uranus and Neptune. For all conditions explored in this work, the mixture behaves as a dense liquid characterized by a diffusive behavior of heavy atoms (Table S2). Observations from our DFT‐MD trajectories revealed a specific pressure–temperature region (22–69 GPa at 3000 K) where water becomes significantly ionized, dissociating into hydronium (H_3_O^+^) and hydroxide (OH^−^) ions.^[^ ^14^, ^15^, ^16^, ^17^ ^]^ In this region the water dissociation monotonously increases as a function of pressure, with values ranging from 8.5 % at 22 GPa to 27.7 % at 69 GPa. This can be appreciated in Figure 1b where the water species populations for all of the four pressure conditions at 3000 K are reported.

Under these conditions (22–69 GPa at 3000 K), frequent proton hopping events among water molecules in the mixture occur along the dynamics, following the Grotthuss mechanism. Notably, methane molecules also take part in these proton transfer chains, becoming protonated by either hydronium ions (Equation 1) or water molecules (Equation 2), leading to the formation of transient methanium cations (CH_5_^+^). The mechanism, along with the Wannier centers associated with the reactive structures, of the proton‐hopping mechanism between hydronium ion and methane (Equation 1) is depicted in Figure 1c. The CH_5_^+^ species exhibit a three‐center two‐electron bond, as can be seen from the corresponding Wannier centers in structure II. The three‐center two‐electron bond is typical of CH_5_^+^ species, previously characterized in gas phase and in superacids through infrared spectroscopy and ab initio calculations.^[^ ^52^, ^53^, ^54^, ^55^, ^56^ ^]^

**Figure 2:**
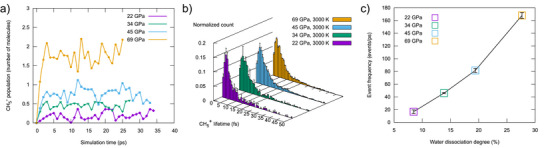
Panel a: The population of CH_5_^+^, averaged over 1 ps intervals, as a function of simulation time at 3000 K within the 22–69 GPa pressure range. Panel b: CH_5_^+^ lifetime distributions in the 22–69 GPa range at 3000 K. Panel c: CH_5_^+^ formation frequency as a function of water dissociation degree. For Panel b,c: error bars correspond to a 95% confidence interval.

We computed the lifetimes of CH_5_^+^ species from the DFT‐MD trajectories, and their distributions across the 22–69 GPa range at 3000 K are reported in Figure 2b. The CH_5_^+^ cation is short‐lived, with an average lifetime of 7.0 fs. This value is slightly longer than that of H_3_O^+^ (6.3 fs), which value is in agreement with previously reported H_3_O^+^ lifetimes in water under extreme conditions.^[^ ^7^, ^16^ ^]^ Although the average lifetime of CH_5_^+^ is short, many species persist for 10–55 fs during the simulations (see distribution in Figure 2b), exhibiting the characteristic hydrogen‐scrambling dynamics, as illustrated in the video of a long‐lived (55 fs) species in the Supplementary Information.

While individual CH_5_^+^ species are in average short‐lived, their overall population remains stable. This can be seen in Figure 2a, which shows the evolution of the CH_5_^+^ populations over the simulation time. After a rapid equilibration, the CH_5_^+^ populations remain stable over the 25–35 ps of DFT‐MD simulations. The long‐term stabilities of CH_5_^+^ population over 200 ps have been assessed by MLIP‐MD (Figure S15a and Section “Machine Learning Interatomic Potentials for CH_5_^+^ formation” of the main text).

The presence of short molecular lifetimes and stable populations indicates the establishment of a fast chemical equilibrium involving the exchange of protons among methane, the methanium cation (CH_5_^+^), water, and hydronium ions in the mixture.

(1) $$H_{3}O^{+}+{\mathrm{CH}}_{4}⇌H_{2}O+{\mathrm{CH}}_{5}^{+}.$$

(2) $$H_{2}O+{\mathrm{CH}}_{4}⇌{\mathrm{OH}}^{-}+{\mathrm{CH}}_{5}^{+}.$$

We quantified the rate of methane protonation over the 22–69 GPa pressure range by computing the frequency of CH_5_^+^ formation at 3000 K as a function of the water dissociation degree (see Figure 2c). The rate is defined as the number of CH_5_^+^ formation events per picosecond. The analysis presented in Figure 2c highlights a direct correlation between the CH_5_^+^ rate of formation and the degree of water dissociation as pressure increase. As water dissociates under the effect of growing pressure, the CH_5_^+^ formation frequency increases almost linearly.

**Figure 3:**
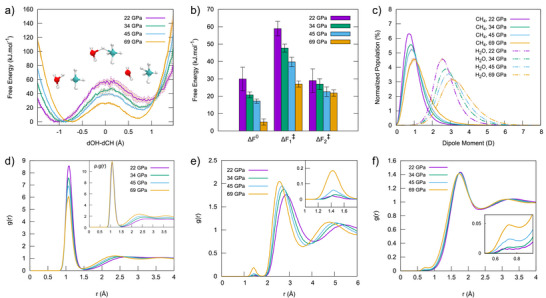
Panel a: Free energies of CH_5_^+^ formation as a function of the reaction coordinate, defined as *d*(O─H) − *d*(C─H), the difference between the distance from the hydrogen to the nearest oxygen and the distance from the same hydrogen to the nearest carbon atom. Panel b: Histogram plot showing the free energy barriers ($\Delta F_{1}^{‡}$ for the forward reaction, $\Delta F_{2}^{‡}$ for the backward reaction) and the reaction free energy (Δ*F*). Panel c: Population of the molecular dipole moments of CH_4_ and H_2_O in the 22–64 GPa range at 3000 K. Panel d: C─H radial distribution functions in the 22–64 GPa range at 3000 K. In the inset the RDFs are multiplied by the corresponding density to show absolute counts. Panel e: Combined C─O and C─C radial distribution functions in the 22–64 GPa range at 3000 K. Panel f: H─H radial distribution functions in the 22–64 GPa range at 3000 K. For Panels a,b,c: All values are accompanied by an error bar corresponding to a 95% confidence interval.

To get a deeper insight into the thermodynamics and kinetics of CH_5_^+^ formation, we present in Figure 3a,b the converged free energy profiles obtained from independent unbiased DFT‐MD simulations (two for P,T condition), associated with the protonation of methane by a hydronium ion (Equation 1) at 3000 K across the pressure range of interest. The free energies, with statistical errors reported at a 95% confidence interval, are directly computed from the DFT‐MD trajectories, fully accounting for the effects of temperature and solvation (see the Method section, Section S1).

From 22–69 GPa, the protonation of CH_4_ becomes kinetically favored, as evidenced by an approximate 50% reduction in the free energy barriers ($\Delta F_{1}^{‡}$). Interestingly, the free energy profiles also show a reduction of Δ*F* (*F* 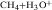 ‐ *F* 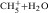) by more than threefold: the reaction is therefore thermodynamically favored with pressure increase, in agreement with the increase of CH_5_^+^ average population with pressure, reported in Figure 2a.

The free energy profiles associated to the CH_5_^+^ formation from water and methane (see Equation 2) are reported in Figure S4. The difference in Δ*F* between the two channels of CH_5_^+^ formation makes the pathway through Equation 1 the most favorable one, irrespective of the pressure. It is worth noting that, despite such difference in Δ*F*, the CH_5_^+^ formation is observed from both H_3_O^+^ and H_2_O. Both channels are thermally activated at 3000 K and occurs at a very fast rate, as confirmed by the small statistical errors of the respective free energy profiles.

As pressure increases, the observed reduction in free energy barriers for both reaction channels aligns with the corresponding rise in the dipole moment of methane. This can be appreciated in Figure 3c, where the instantaneous dipole moment of methane, calculated using Wannier centers,^[^ ^57^ ^]^ is presented at 3000 K within the pressure range of 22–69 GPa. The increase in pressure causes methane molecules to exhibit an instantaneous dipole moment, with the maximum absolute value progressively increasing from 0.73 D at 22 GPa to 1.00 D at 69 GPa. Additionally, with rising pressure, the distributions widen, indicating a growing number of methane molecules with elevated dipole moments in the simulation. The dipole moment induced by pressure enhances the proton affinity of CH_4_ molecules, thereby favoring their protonation.

To investigate the role of water in this phenomenon, we show in Figure S5, a comparison of the dipole moment distributions for pure liquid CH_4_ (red) and the CH_4_/H_2_O mixture (blue). At 47 GPa and 3000 K, methane exhibits a significantly lower dipole moment in the pure liquid than in the mixture under similar P,T conditions. These findings suggest that, in addition to high pressure and temperature, the pronounced ionization and polarization of water under extreme conditions play a key role in enhancing the CH_4_ dipole moment, thereby favoring its protonation.

In Section S7, we report the observation of non‐classical CH_5_^+^ species at dilute solution conditions (1 methane for 127 water molecules) and lower temperature (2000 K). This establishes the presence of pentacoordinated CH_5_^+^ species in aqueous solutions across a broad range of pressures, temperatures, and methane concentrations.

The molecular species sampled along the MD trajectories were identified using a molecular recognition algorithm based on distance criteria (see Section S8 for details). For the CH_5_ structures, we verified that the identified species correspond to CH_5_^+^ rather than CH_5_^−^ by comparing the results obtained from the distance‐based criterion with those derived from a combined distance‐ and charge‐based analysis employing Wannier centers. The excellent agreement between the two approaches (see Table S3) confirms that the CH_5_ structures identified by the distance‐based algorithm correspond to positively charged CH_5_^+^ species. Furthermore, we verified that the identified CH_5_^+^ species predominantly exhibit three‐center two‐electron bonding, characterized by Wannier centers displaced from the C─H bonds and located mainly in the middle of the H─C─H angles. In addition, the CH_5_^+^ species display asymmetric C─H distance distributions, and longer C‐H bonds with respect to CH_4_, typical of CH_5_^+^ structures reported in the literature. All these analysis are reported and discussed in details in Section S8.

The increasing formation of pentacoordinated CH_5_^+^ species, characterized by longer C─H distances, with pressure is reflected in the C─H radial distribution functions reported in Figure 3d. The rising intensity at the first minimum indicates the formation of longer C─H bonds at increasing pressure. In the inset, the RDFs are multiplied by the corresponding density to show absolute counts, highlighting the increase at the first minimum even more clearly.

### The Fate of CH_5_^+^: Superacid‐Like Oligo‐Condensation of Methane

Our focus now turns to the reaction mechanisms that underlines the condensation of methane in water, with a particular focus on the possible role of pentacoordinated carbonium ions (such as CH_5_^+^) in these transformations. For instance, the DFT‐MD simulations at 3000 K reveal that methanol and ethane are formed across the entire pressure range (22–69 GPa), while longer hydrocarbons such as propane and butane are only formed at 45 and 69 GPa, respectively. The spontaneous formation of C─C and C─O bonds is clearly captured in the radial distribution functions (Figure 3e), where the appearance of a peak at 1.4 Å  intensifies with increasing pressure. Additionally, the formation of H_2_ molecules is also observed, as reflected in the corresponding radial distribution functions (Figure 3f), where a peak emerges at 0.7 Å and grows with increasing pressure.

Four water‐mediated catalytic mechanisms are identified: three acid‐catalyzed and one base‐catalyzed. Notably, all three of the acid‐catalyzed pathways involve the formation of pentacoordinated carbonium species, like CH_5_^+^. Once formed, the carbonium ions either undergo deprotonation, which accounts for hydrogen exchange with water, or they react to form C─C and C─O bonds via one of the three acid‐catalyzed pathways.

#### M1 Mechanism

**Figure 4:**
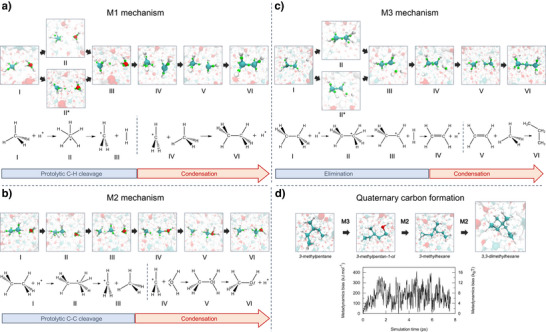
Scheme of the reaction mechanisms, along with the Wannier centers associated with the reactive structures for the superacid M1 (Panel a) and M2 (Panel b), as well as the M3 superacid catalyzed reaction mechanism (Panel c). Panel d: scheme of the reaction mechanism (top) and associated metadynamics bias (bottoms) for quaternary hydrocarbon species formation at 3000K and 50 GPa.

The first acid‐catalyzed mechanism, M1, is an elongation reaction where a C─C or C─O bond is added to an alcohol or hydrocarbon chain. In this mechanism, the pentacoordinated carbonium ion undergoes dehydrogenation, generating a highly reactive trivalent carbenium ion (e.g., CH_3_^+^, CH_3_CH_2_^+^). This undercoordinated species then reacts with water or methane, leading to the formation of alcohols or hydrocarbons, respectively. Figure 4a illustrates ethane formation from methane. The reaction is initiated by the protonation of methane by a hydronium ion or water molecule, resulting in a CH_5_^+^ structure characterized by a two‐electron, three‐center C─H_2_ bond (structure II). The reaction proceeds with the cleavage of the C─H_2_ bond, release of H_2_ into the solution, and the formation of the highly reactive CH_3_^+^ ion. Finally, ethane or methanol molecules are formed through an electrophilic attack by the CH_3_^+^ ion on a neighboring methane or water molecule, along with the concurrent release of a proton. As observed in the DFT‐MD simulations, the same M1 mechanism also leads to the formation of propane (45 GPa) and butane (69 GPa). In these cases, the reaction proceeds either through the formation of CH_5_^+^ or more complex pentacoordinated carbonium ions (e.g. CH_3_CH_4_^+^, CH_3_CH_2_CH_4_^+^); their dehydrogenation follows, along with the formation of highly reactive trivalent carbenium ions (CH_3_CH_2_^+^, CH_3_CH_2_CH_2_^+^). The M1 mechanism can also proceed through the formation of a slightly different CH_5_^+^ geometry (structure II*),^[^ ^47^, ^58^ ^]^ observed at 3000 K, as reported in Figure 4a. In this case, the reactive electronic doublet is displaced close to the reactive C─H hydrogen, ready to be caught by the incoming H^+^. The proton attacks the hydrogen atom of methane, leading to an unstable CH_5_^+^ geometry. Here, the reaction occurs “on the fly” without passing through a three‐center two electron bond CH_5_^+^ species.

Remarkably, the M1 mechanisms revealed by our simulations correspond to the mechanisms experimentally characterized for the oligomerization of methane in superacid liquid at moderate temperatures (60–150 °C) and ambient pressure, as reported in the landmark studies by Olah et al.^[^ ^47^, ^48^ ^]^ In these transformations, the condensation of CH_4_ in strong superacids (e.g. SbF_5_/HF, approximately 10^9^ times stronger than pure sulfuric acid) is driven by the formation of pentacoordinated carbonium ions such as CH_5_^+^ and RCH_4_^+^, which undergo subsequent dehydrogenation. This process leads to the generation of highly reactive trivalent carbocations (e.g., CH_3_^+^, RCH_2_^+^), triggering hydrocarbon condensation reactions. It is important to note that the short average lifetime of CH_5_^+^ observed in our simulations of CH_4_/H_2_O under extreme conditions (see Figure 2b) is consistent with the well‐known short‐lived nature of CH_5_^+^ in superacid liquids, where AIMD simulations^[^ ^54^ ^]^ and experimental studies^[^ ^59^ ^]^ have shown that CH_5_^+^ species are fleeting intermediates, highly unstable, existing in the femtosecond timescale.^[^ ^54^ ^]^

#### M2 Mechanism

The second acid‐catalyzed reaction mechanism (M2) accounts for the interconversion of alcohol to alkanes and vice versa, as well as between different alkanes. In this mechanism, pentacoordinated carbonium ions (e.g., CH_3_─CH_4_^+^) or protonated alchool (e.g. CH_3_OH_2_^+^) evolve respectively through the cleavage of C‐C and C‐O bonds, forming highly reactive trivalent carbenium ions (e.g., CH_3_^+^). The carbenium ions then react with nearby water or methane to form new C─C or C─O bonds.

An example of M2, observed along the DFT‐MD, is illustrated in Figure 4b for the interconversion of ethane to methanol. The reaction starts with protonation of ethane by a hydronium ion or water molecule, forming a pentacoordinated CH_3_CH_4_^+^ ion. The reaction then proceeds through the protolytic cleavage of the C─C bonds and results in the formation of the reactive CH_3_^+^ ion. As in the case of the M1 mechanism, ethane or methanol molecules are formed through an electrophilic attack by the CH_3_^+^ ion on a neighboring methane or water molecule. It is important to highlight that the formation of ethane from methanol (opposite reaction of the one in Figure 4c) through the formation of CH_3_OH_2_^+^ cation is also observed. This transformation is important because it converts alcohols to alkanes (C─O → C─C), showing that alcohol formation is not a dead end for hydrocarbon chain elongation in water. The M2 mechanism has been proposed, alongside the M1 mechanism, by Olah et al.^[^ ^60^ ^]^ for alkane polycondensation and isomerization in strong superacids. This parallel further confirms the superacid‐like hydrocarbon condensation mechanisms in water under extreme conditions.

#### M3 Mechanism

The third acid‐catalyzed mechanism, M3, is an elongation reaction in which pentacoordinated carbonium ions convert into double‐bonded intermediates, such as ethylene. Figure 4c illustrates the formation of propane via the M3 pathway. Ethane is first protonated by H_3_O^+^ or water, forming a pentacoordinated CH_3_CH_4_^+^ species. The intermediate then releases molecular hydrogen (H_2_), yielding the under‐coordinated CH_3_CH_2_^+^ cation. Unlike in the M1 mechanism, the trivalent CH_3_CH_2_^+^ species undergoes proton elimination to form ethylene (lifetime in Figure S12). The ethylene then reacts via electrophilic attack on a nearby methane or water molecule, yielding propane or ethanol. Interestingly, Olah et al. have proposed the same mechanism for the formation of ethylene from C_2_H_7_^+^ ion in strong superacids.^[^ ^60^ ^]^ However, in superacid solutions, unlike in our M3 mechanism where ethylene reacts further with methane or water, it is proposed that ethylene primarily react with carbenium ions such as CH_3_^+^ or CH_3_CH_2_^+^. We also observe a water base‐catalyzed variant of the M3 mechanism, as reported and discussed in Figure S11. In this variant, the double‐bonded intermediate originates from an anionic species formed via the deprotonation of a hydrocarbon or alcohol by an OH^−^ species.

#### Methane Oligo‐Condensation in Water and in Superacids

Methane oligo‐condensation in water under extreme conditions proceeds via acid‐catalyzed reaction mechanisms analogous to those characterized for the poly‐condensation of methane in strong superacids close to ambient conditions. Despite these mechanistic parallels, there are also differences between the two chemistry. In water, the high OH^−^ concentration open the way for hydrocarbon condensation via base‐catalyzed pathways (Figure S11), which are obviously absent in superacids. The absence of water in superacids also exclude C─O bond formation and the associated chemistry. The chemical and physical origins of CH_5_^+^ species in water and in superacids are different. In superacid solutions (e.g., SbF_5_/HF), the combination of strong Lewis and Brønsted acids is proposed to induce a high proton chemical potential in solution, enabling protonation of the weak base CH_4_. In water, by contrast, CH_5_^+^ formation arises directly from extreme temperature and pressure. High pressure shifts the thermodynamic equilibrium toward CH_5_^+^ by reducing Δ*F* for its formation (Figure 4a,b) and increases the concentration of H_3_O^+^, the reactant in the most favorable protonation pathway (Equation 1). Elevated temperature further promotes CH_5_^+^ formation by flattening the Boltzmann distribution, thereby shifting the equilibrium constant toward formation of CH_5_^+^.

### Hydrocarbon Branching and Quaternary Carbon Structures Formation

In the previous sections, we provided a mechanistic rationalization of the early stages of hydrocarbon chain elongation in water. Here, our aim is to determine whether the mechanisms and chemistry identified for the earlier stages are also at play in the hydrocarbon branching and quaternary carbon structure formation.

To achieve this, we performed metadynamics simulations, biaising along a topologically‐aware collective variable knicknamed SPRINT,^[^ ^61^ ^]^ which is a general‐purpose CV designed to explore the possible arrangements of a system's bond network. Using these coordinates, we first performed a series of six independent metadynamics simulations of water/methane mixtures using systems of 488 atoms at 3000 K and 45 GPa. By examining the maximum value of the metadynamics energy bias introduced during the simulations, we obtain an upper bound of the free energy barriers associated with these transformations, thereby assessing their feasibility. On the limited timescale of the metadynamics (17 ps maximum), three of the six simulations led to the formation of complex branched hydrocarbon species containing secondary and tertiary carbon centers (i.e. carbons bonded to two and three other carbons, respectively). Crucially, all the simulations followed the mechanism identified in the previous sections (M1, M2, and M3) and revealed free energy barriers of less than 27.8 *k*_*B*_ *T*. These results demonstrate the favorability of hydrocarbon chain elongation and branching, extending the non‐classical carbocation chemistry in water under extreme conditions discussed earlier.

Next, we investigated the formation of hydrocarbon structures bearing quaternary carbon atoms (i.e. carbons bonded to four other carbons), reminiscent of diamond structures. For this purpose, we performed metadynamics on a larger system composed of 722 atoms, containing H_2_O/CH_4_ and a 3‐methylpentane molecule previously obtained in our smaller‐scale simulations, the detailed mechanism of its formation being reported in the Figure S14. As illustrated in Figure 4d, at 3000 K and 45 Gpa, 3‐methylpentane evolved into 3,3‐dimethylhexane, a quaternary carbon structure, following the acid M3 and M2 pathways. Importantly, the metadynamics bias never exceeded 16 *k*_*B*_ *T* (see Figure 4d), indicating that these transformations are energetically accessible under these conditions.

Reinforcing this observation, an unbiased DFT‐MD simulation initially containing a tertiary hydrocarbon species revealed the spontaneous formation of a quaternary alcohol, which subsequently interconverted into quaternary hydrocarbon species via a acid‐catalyzed M2 mechanism within a few picoseconds (Figure S13). The free energy barrier associated with quaternary carbon formation at 3000 K and 45 GPa is therefore probably much lower than the estimated upper bound from metadynamics. Finally, we confirmed the metastability of the 3,3‐dimethylhexane (last structure in Figure 4c) by performing six unbiased MD simulations. We observed that the carbon backbone remains intact for more than 5.5 ps in three of the six simulations at 3000 K and 45 GPa. By contrast, frequent hydrogen exchanges occur between the C─H groups of the hydrocarbon structure and water species in the surrounding.

We note that our dynamics do not include the effect of zero‐point energy (ZPE), which could influence the stability of the C─C bonds in the hydrocarbon species studied here. Future simulations incorporating nuclear quantum effects will help clarify the potential impact of ZPE on hydrocarbon condensation reactions in water.

**Figure 5:**
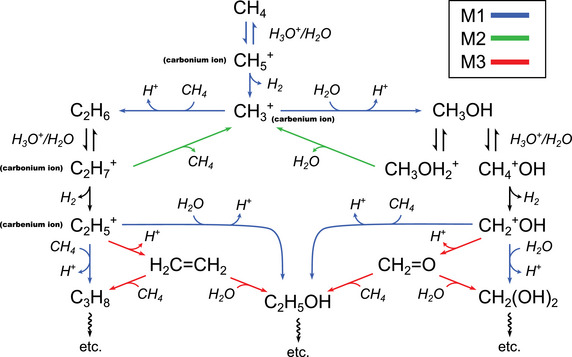
Scheme of the reaction network for water acid‐catalyzed hydrocarbon and alcohol condensation. Blue/Red arrows: elongation and branching reactions through either the M1 or M3 mechanisms. Green arrows: alcohol/alkane interconversion reactions through the M2 mechanism.

By distilling the results presented above, we are now able to present, in Figure 5, a first picture of the water acid‐catalyzed hydrocarbon condensation at HPHT conditions as a reaction network. Initially, CH_4_ condenses exclusively via the acid catalyzed M1 mechanism (blue pathways), through the transient formation of CH_5_^+^ species, leading to ethane or methanol. Ethane and methanol undergo proton exchange with the water medium, leading to the formation of pentacoordinated carbonium ions (C_2_H_7_^+^ and CH_4_OH^+^). These intermediates subsequently release H_2_, producing highly reactive trivalent species (C_2_H_5_^+^ and CH_2_OH^+^). If the trivalent species undergoes a proton elimination, hydrocarbon chain elongation proceeds via the M3 mechanism (green pathways) through the formation of double‐bond intermediates (CH_2_═CH_2_ or CH_2_═O). Alternatively, if the trivalent species condense directly with methane or water, the reaction follows the M1 mechanism (blue pathways). Alcohols and alkanes can inter‐convert at different stages of the process, via the acid‐catalyzed M2 mechanism (green pathways). As molecular complexity increases, hydrocarbon chains extend, branch and isomerizes through the same M1, M2, and M3 pathways, as observed in metadynamics and unbiased DFT‐MD simulations. This framework, unveiled in our study, deciphers the step‐wise transformation of methane in ionized water, revealing the core chemistry driving the hydrocarbon condensation under extreme conditions.

### Machine Learned Interatomic Potentials for CH_5_^+^ Formation

**Figure 6:**
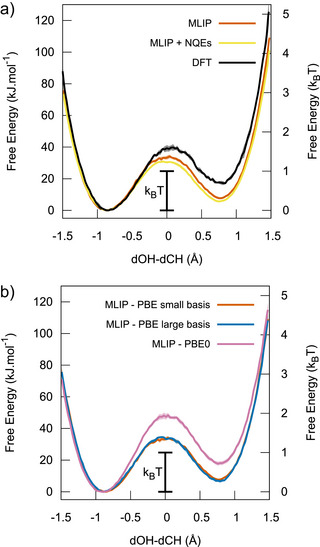
Panel a: Comparison between free energy profiles obtained through DFT‐MD (black line), and with a surrogate MLIP model through classical molecular dynamics (red line) or with the inclusion of nuclear quantum effects (yellow line). Panel b: Free energy profiles comparing different MLIPs trained on three datasets: DFT‐MD settings (”PBE small basis”, red line), larger basis set (”PBE large basis”, blue line), and a hybrid functional (”PBE0”, pink line). All values are accompanied by an error bar corresponding to a 95% confidence interval.

We then assessed the impact of our computational setup on our results regarding the formation of CH_5_^+^ in water. Since GGA functionals are known for their inaccuracies on reaction barriers and energies,^[^ ^62^, ^63^ ^]^ we have evaluated the consistency of our results using a hybrid functional. In addition, we tested the possible influence of basis set size, as well as nuclear quantum effects. For such tests to be affordable, we have implemented surrogate models at 45 GPa in the form of machine‐learned interatomic potentials^[^ ^46^ ^]^ (MLIPs), their training starting from the configurations sampled through DFT‐MD. The results are presented in Figure 6a,b.

First, although the agreement is not perfect, with some overstabilization of the CH_5_^+^ state and acceleration of the kinetics, the MLIP surrogate satisfyingly reproduces the thermodynamics and kinetics of proton transfer involving H_3_O^+^ and CH_5_^+^ at the PBE level. Indeed, due to the high thermal energy, the computed values of Δ*F* ^0^ and $\Delta F_{1}^{‡}$ differ from the DFT results by less than 1 and 0.3 *k*_*B*_ *T*, respectively. We used the surrogate model to assess the convergence of the free energy profiles and the stability of CH_5_^+^ population over longer simulation time. As can be seen in Figure S15a, the MLIP free energy remains stable at 45 GPa and 3000 K over 200 ps. Second, we employed path‐integral molecular dynamics (PIMD) using the surrogate model to assess the relevance of nuclear quantum effects. While elevated temperatures typically reduce the impact of quantum fluctuations, extreme pressures can enhance them.^[^ ^64^ ^]^ Nonetheless, nuclear quantum effects were found to be negligible in this case, with reaction free energies changing by less than 0.1 *k*_*B*_ *T* (see orange and yellow curves in Figure 6a). Third, basis set size is shown to have no effect on the reaction we monitor, with both free energy profiles associated with small and large basis sets being superimposed (see orange and blue curves in Figure 6b). Finally, switching to a hybrid functional has a slightly larger effect, since the CH_5_^+^ state is destabilized by less than 0.5*k*_*B*_ *T*, and the barrier increases by 0.6*k*_*B*_ *T*. The effects remain however marginal, since the absolute barrier at the hybrid level is low (less than 2*k*_*B*_ *T*) which means that the reaction remains thermally activated at 3000 K and occurs at a very fast rate. This further reinforces our claim that the formation of non‐classical CH_5_^+^ ions takes place in CH_4_/H_2_O mixtures at extreme conditions by showing that it is not strongly altered by a change in DFT functional.

## Conclusions

We show, for the first time, that transient hypercoordinated carbocations, like CH_5_^+^, emerges in ionized water under extreme thermodynamic conditions and govern the hydrocarbon condensation process. To demonstrate this phenomenon we integrated DFT, machine learning, and advanced enhanced sampling techniques. Our results reveal that, as pressure and temperature increase, the methane molecule amplifies its dipolar response within an increasingly ionized and polarized water medium. This favors the thermodynamic and kinetic formation of pentacoordinated carbonium ions. These carbonium ions act as key fleeting intermediates in hydrocarbon condensation, being involved in all the acid‐catalyzed hydrocarbons condensation mechanisms. Remarkably, the chemistry we observe closely parallels the superacid‐catalyzed hydrocarbon condensation described in the Nobel Prize–winning work of Olah and coworkers.^[^ ^47^, ^48^ ^]^

These findings open new frontiers in understanding and modeling chemical transformations in aqueous environments at high pressures and temperatures. Remarkably, the capability of water in protonating one of the weakest base, methane, may play a dominant role in environments where water becomes ionized, including Earth's upper mantle, superionic ice, and the recently discovered superionized phase of nanoconfined water.^[^ ^13^ ^]^ Our framework provides a new roadmap for high‐pressure, high‐temperature synthesis routes of hydrocarbons and nanodiamonds precursors, with significant implications for petrochemistry, geochemistry, and advanced materials synthesis. Importantly, these findings also bear relevance for planetary science, offering key input for thermodynamic and kinetic models of chemical evolution in dense C/H/O mixtures within the interiors of icy giants and sub‐Neptune planets.

As a future perspective, identifying computational IR and Raman markers of CH_5_^+^ in water under extreme conditions could enable its detection in laser‐heated diamond anvil cell (LH‐DAC) experiments. The computed vibrational density of states of CH_5_^+^ in water (Figure S10b) shows qualitative band shifts relative to CH_4_, suggesting that vibrational spectroscopy may provide a feasible route for the experimental identification of carbonium ions in water under extreme conditions.

## Conflict of Interests

The authors declare no conflict of interest.

## Supporting information



Supporting Information

Supplemental Video 1

## Data Availability

The data that support the findings of this study are available in the supporting information of this article.
