# Supplementary material for: From Methane to Nanodiamond Precursors in Water: Superacid‐like Condensation Pathways Under Extreme Conditions
Source: Angew Chem Int Ed Engl. 2025 Nov 26;65(3):e20364. doi: 10.1002/anie.202520364 (PMC12811670; doi:10.1002/anie.202520364)
Supplement: Supplementary file 1 — Supporting Information [file ANIE-65-e20364-s002.pdf]

1. Methods

DFT-MD simulations

All DFT-MD simulations relied on the Born-Oppenheimer approximation, and were performed in the canonical (NVT) ensemble with a time step of 0.5 fs, using a Nosé-Hoover chain of three thermostats with a time constant of 50 fs. Starting configurations were generated using PACKMOL [1]. For each P–T conditions, two independent dynamics ranging from 25 to 42 ps have been performed using simulation boxes of 488 atoms (52 CH_4 and 76 H_2O molecules). All the analysis and associated error bars are performed on these trajectories, excluding the first 7 ps of equilibration. Larger simulation boxes (722 atoms) were adopted to probe the stability and reactivity of complex hydrocarbon species featuring tertiary and quaternary carbon centers.

All DFT-MD simulations were performed using the CP2K package [2], under the generalized gradient approximation (GGA), with the PBE functional [3] and D3 Becke-Johnson dispersion corrections [4]. We solved the spin-restricted Kohn-Sham equations self-consistently using the orbital transformation method, with an associated convergence criterion set to 10^{-6} Ha. The electronic density and wavefunction are represented using a dual basis of atomic orbitals and plane waves, as implemented in QUICKSTEP [2]; we truncate the plane wave expansion using a 950 Ry cutoff, and use DZVP-MOLOPT-SR basis sets. Supplementary Section 14 describes the details of the DFT calculation performed for MLIP labelling with different functionals and basis sets. Metadynamics-accelerated DFT-MD simulations have been performed in order to enhance the sampling of the H_2O/CH_4 mixture phase space toward hydrocarbon branching and diamond-like structure formation. Details regarding metadynamics simulations and the definition of the SPRINT reaction coordinates are presented in Supplementary Sections 12 and 13.

Free energies from unbiased DFT molecular dynamics

The free energy $F(x)$ as a function of the reaction coordinate x, reported in Figure 3a of the Main Text, is computed directly from the DFT-MD trajectories using the expression from statistical mechanics:

$$F(x) = -k_B T \log P(x) \tag{1}$$

where $P(x)$ is the probability distribution of locating the transferring hydrogen atom at a given value of the reaction coordinate x. The reaction coordinate is defined as the difference between the hydrogen–oxygen and hydrogen–carbon first-neighbor distances:

$$x = d_{\mathrm{H-O}} - d_{\mathrm{H-C}} \tag{2}$$

The oxygen and carbon atoms belong to the reactive species (identified by the molecular recognition program, described in Supplementary Section 8) involved in the reaction under consideration, e.g., CH_4, H_3O^+, CH_5^+, and H_2O in the case of reaction 1 (protonation of methane from a hydronium ion). The free energy profiles for CH_5^+ formation (see Figure 3a and Figure S4) are converged, as can be seen from the small statistical error (95% confidence interval) estimated from independent dynamics.

Recognition Program

Details on the molecular recognition analysis implemented in this study to compute the population of species (Figure 2a in the main text) and CH_5^+ rate of formation (Figure 2c in the main text) are reported in Supplementary Section 8.

MLIPs: training and MD

All the details on the MLIP training and MLIP-MD silmulations can be found in Supplementary Sections 15 and 16.

2. Electronic density of states

In Figure S1, we report the electronic density of states (DOS) for the CH_4/H_2O mixture in the 22–69 GPa range at 3000 K, obtained by averaging, for each P, T condition, the DOS of 200 frames from DFT-MD simulations. A sizable band gap is observed across all systems, showing showing a slight dependence on pressure. The DOS at 45 GPa and 3000 K agrees well with that reported by Lee et al. [5], who studied a smaller CH_4/H_2O system of the same stoichiometry using plane-wave DFT as implemented in the Quantum Espresso program. In order to better

evaluate the magnitude of the band gap we report in Table S1 the difference HOMO-LUMO averaged over 200 frames in the 22-69 GPa range.

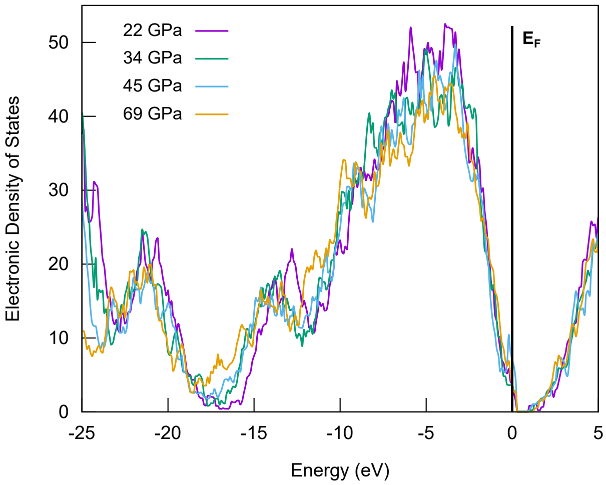

Figure S1. Electronic density of states in the 22-69 GPa range at 3000 K. E_F indicates the Fermi energy. The purple line corresponds to 22 GPa, the green line corresponds to 34 GPa, the blue line corresponds to 45 GPa and the yellow corresponds to 69 GPa. All the systems were simulated at 3000 K.

Table S1. The first column displays the pressure conditions. Columns two reports the HOMO-LUMO band gap (ev) averaged over 200 structures extracted from the DFT-MD trajectories

P (GPa)	HOMO - LUMO gap [eV]
22	2.02 ±0.76
34	1.87 ±0.81
45	1.87 ±0.67
69	1.68 ±0.77

3. O-H radial distribution functions

In Figure S2, we report the O–H radial distribution function (RDF) for the CH_4/H_2O system in the 22–69 GPa range at 3000 K. The RDF analysis clearly indicates an increasing degree of water dissociation, as evidenced by the significant intensity of the O–H RDF near its first minimum. A similar increase in the first O–H RDF minimum with pressure was also observed for pure liquid water at 1000 K in the 11–20 GPa range by Rózsa et al.[6], which was attributed to enhanced water dissociation. It should be noted that the decrease in the intensity of the first peak at higher pressures is simply due to the increasing system density, as the RDF is normalized by the density of the system.

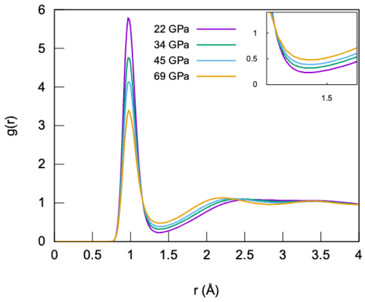

Figure S2. O-H radial distribution functions for the CH_4/H_2O system in the 22-69 GPa range at 3000 K.

4. H_3O^+ lifetime distributions

In Figure S3 we report the lifetime distributions for H_3O^+ in the range 22-69 GPa at 3000K, calculated from DFT-MD simulations through the molecular recognition program.

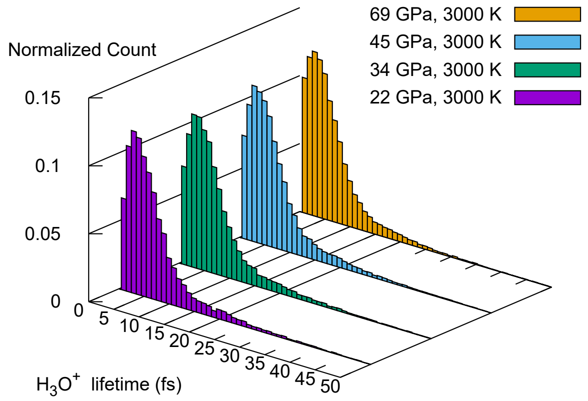

Figure S3. H_3O^+ lifetime distributions in the 22-69 GPa range at 3000 K.

5. Free energy profile for CH_5^+ formation from CH_4+H_2O

In Figure S4 we report the free energy profiles for the CH_5^+ formation from the CH_4+H_2O reaction channels at 3000 K in the pressure range 22-69 GPa. Despite the more than twofold increase in free energy barriers compared to the reaction involving methane and hydronium, the pressure-dependent decrease in free energies follows a qualitatively similar trend.

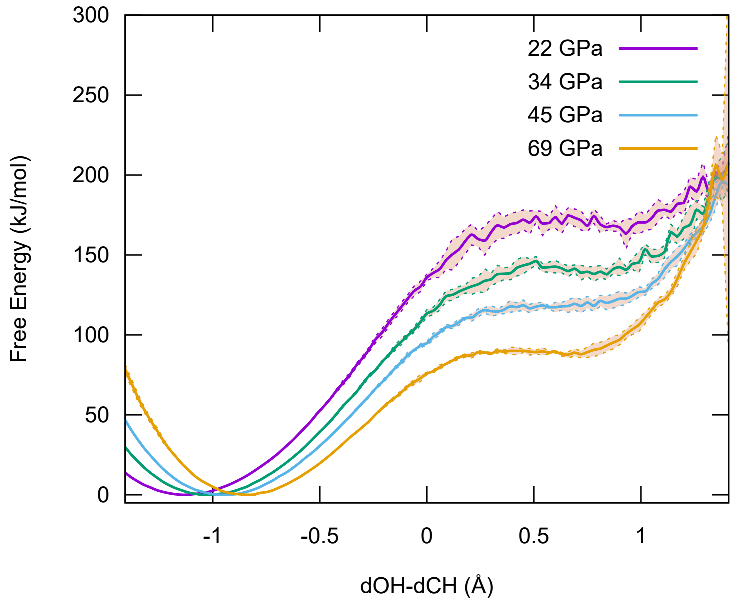

Figure S4. Free energy of CH_5^+ formation from CH_4+H_2O as a function of the reaction coordinate, defined as d(O-H) $-$ d(C-H), the difference between the distance from the hydrogen to the nearest oxygen and the distance from the same hydrogen to the nearest carbon atom. All values are accompanied by an error bar corresponding to a 95% confidence level.

Table S2. The first column displays the pressure conditions. Columns two, three and four report the diffusion coefficients at 3000 K for the different atom kinds.

P (GPa)	D_O (m^2/s)	D_C (m^2/s)	D_H (m^2/s)
22	2.7987×10^{-8}	2.1921×10^{-8}	2.6425×10^{-8}
34	1.9185×10^{-8}	1.3221×10^{-8}	2.3571×10^{-8}
45	1.6707×10^{-8}	1.4959×10^{-8}	2.6346×10^{-8}
69	0.9240×10^{-8}	0.7748×10^{-8}	2.5878×10^{-8}

6. Effect of water on CH_4 dipole moments

We show in Figure S5 a comparison of the dipole moment distributions for pure liquid CH_4 (red) and the CH_4/H_2O mixture (blue). At 47 GPa and 3000 K, methane exhibits a significantly lower dipole moment in the pure liquid than in the mixture under similar P,T conditions.

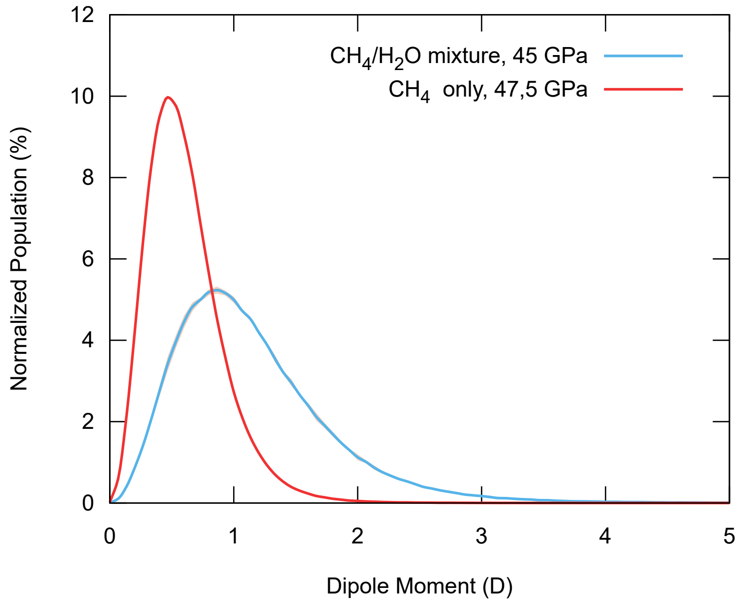

Figure S5. Population of the CH_4 molecular dipole moments of CH_4/H_2O mixtures (blue) and pure liquid methane (red) at 45 GPa and 3000K. The box of pure methane is composed by 475 atoms (95 methane molecules), simulated for 22 ps by GGA-DFT-MD. All values are accompanied by an error bar corresponding to a 95% confidence interval.

7. CH_5^+ formation in the 2000-3000 K range for low CH_4 concentrations

To investigate whether CH_5^+ formation[7–13] persists in water at lower CH_4 concentrations and below 3000 K, we performed two independent DFT-MD simulations of a water–methane mixture composed by 1 methane and 127 water molecules at 45 GPa and at temperatures of 2000 and 3000 K, respectively. In both simulations, the methane molecule participates in proton-hopping mechanisms with water and H_3O^+. The methane protonation mechanism from water, along with the Wannier centers associated with the reactive structures, is illustrated in Figure S6 for the reaction at 2000 K and 45 GPa. The methane first accepts a proton (depicted in orange) from a nearby water molecule, forming a $CH_5{}^+$ species (identified by its Wannier centers). Subsequently, it donates a proton (blue) to another water molecule. The structure labeled as II clearly exhibits the two-electron three-center bond[14–21] characteristic of the $CH_5{}^+$ species. This process occurs after 0.5 ps at 3000 K and after 2.5 ps at 2000 K. As already discussed in the main text, the protonation of CH_4 can occur with both H_2O and H_3O^+ species. Our results demonstrate that the formation of CH_5^+ is observed even at 2000 K and 45 GPa, despite the relatively low concentration of CH_4.

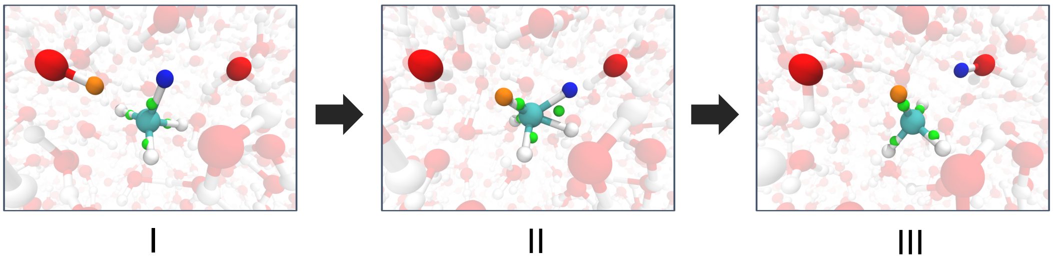

Figure S6. Reaction structures with Wannier centers associated to the Grotthuss-like proton-hopping mechanism between water and methane at 45 GPa and 2000 K. The proton donated by water is shown in orange, while the proton donated by methane is shown in blue.

8. Molecular recognition programs

8.1. Identification of CH_5^+

The molecular recognition method adopted for the population analysis (Figure 1b and Figure 2a,b in the Main Text) and free energy profiles (Figure 3a in the Main Text), is based on the postulate that each hydrogen atom is singly bonded to one heavy atom (carbon or oxygen). Each hydrogen atom is assigned to its first neighboring heavy atom (C or O). This procedure successfully identifies all the relevant molecular species (CH_4, CH_5^+, H_3O^+,OH^-, CH_3^+). However, the possible presence of H_2 molecules in the system, formed as a byproduct of C–C and C–O bond formation reactions, necessitates the introduction of a radial cutoff to exclude these atoms from recognition. To achieve this, we apply radial cutoffs to H–H, C–H, and O–H distances. Specifically, we identify hydrogen atoms that do not fall within the coordination shells of carbon and oxygen, where the shell radii are defined by the respective C–H and O–H cutoffs. Next, we determine whether these hydrogen atoms are linked to other hydrogen atoms that are not associated with heavy atoms, within the proximity defined by the H–H cutoff. If no H–H linkage is detected, the hydrogen atom is assigned to the nearest heavy atom. We assessed the sensitivity of our analysis to the choice of C–H and O–H cutoff values, as illustrated in Figure S9 for four systems at 3000 K in the pressure range of 22–69 GPa. The cutoff values fall within a stable plateau region, confirming the robustness of our method and its independence from the specific C–H and O–H cutoff values. To further validate this, we performed our analysis on parts of the trajectory that do not contain H_2 molecules and compared the results obtained with and without the cutoff. In both cases, the results remained consistent, demonstrating the reliability of our approach.

As a final test, we assessed the performance of the molecular recognition operation by augmenting it with molecular charges from a Wannier center analysis. Species are therefore identified using both the coordination number of heavy atoms and the overall molecular charge. The molecular charges are determined by associating each Wannier center with its nearest neighbor (either a heavy atom or a hydrogen atom from H_2 molecules). In Table S3 we display populations computed with and without the criterion on Wannier centers, using a 13 ps trajectory at 45 GPa and 3000 K. The excellent agreement between both approaches indicates that CH_5 structures identified by the distance based algorithms are CH_5^+ species.

Table S3. Comparison of species populations computed by molecular recognition with Wannier centers (Population 2) and without (Population 1).

Species	Populations 1 (%)	Populations 2 (%)
OH^-	9.518	9.517
H_2O	80.45	80.44
H_3O^+	9.252	9.261
CH_4	88.89	88.89
CH_5^+	1.491	1.475

8.2. Identification of the three-center-two electron bond and CH_5^+ distance analysis

After confirming that the identified CH_5 structures correspond to CH_5^+ species, we analyzed the angles between the Wannier centers and the closest C–H bonds (C–H_1 and C–H_2) to investigate the possible presence of three-center two-electron bonds. The analysis was performed over a 13 ps trajectory obtained at 45 GPa and 3000 K, where the Wannier centers were recorded at each time step.

Figure S7a reports the distribution of θ_1, defined as the angle between the carbon–Wannier center distance

(C–w, shown in the inset) and the nearest C–H bond (C–H_1). For each CH_5^+ species, the distribution of the maximum θ_1 value observed for each identified CH_5^+ species is presented. The data show that all CH_5^+ species exhibit nonzero θ_1 angles, with a maximum around 20°, indicating a significant displacement of the Wannier center from the C–H bond axis, consistent with the formation of a three-center two-electron bond.

To identify the relative position of the three-center two-electron bond electrons with respect to the H_1–C–H_2 angle (θ_2), we report in Figure S7b the distribution of the ratio θ_1/θ_2 computed for each identified CH_5^+ species. The distribution maximum around 0.5, indicates that most CH_5^+ species exhibit a Wannier center located on the bisector of the H_1–C–H_2 triangle, consistent with a three-center two-electron bonds.
As a final analysis, we present in Figure S7c the distribution of C–H distances for the CH_4 (purple curve) and CH_5^+ (red and green curves) species. The C–H distance distributions of CH_5^+ exhibit maxima at longer bond lengths and are broader than that of CH_4, indicating longer C–H bonds. Furthermore, the C–H bonds in CH_5^+ involved in three-center two-electron interactions (red curve) are longer than those that are not (green curve), in agreement with the CH_5^+ structures reported in gas-phase studies in the literature.[11,18,21,22]

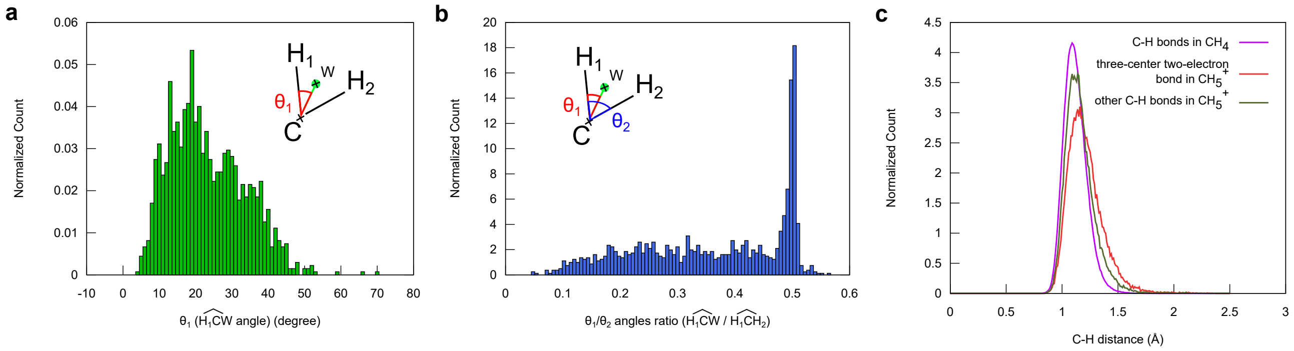

Figure S7. Panel a: Distribution of the maximum θ_1 angles experienced for each CH_5^+ species identified along a 13 ps simulation at 3000 K and 45 GPa. Here, θ_1 is defined as the angle between the carbon–Wannier center vector (C-w, shown in the inset) and the nearest C–H bond (C–H_1, shown in the inset). **Panel b**: Distribution of the ratio θ_1/θ_2 for each CH_5^+ species identified. θ_1 is the angle between the carbon–Wannier center vector (C–w) and the nearest C–H bond (C–H_1, red in the inset), while θ_2 is the angle between the two C–H bonds closest to the Wannier center (C–H_1 and C–H_2, angle reported in blue in the inset). **Panel c**: Distribution of C–H distances from the 13 ps DFT-MD simulation at 45 GPa and 3000 K. The purple line shows the C–H bond distribution for CH_4 species. The green and red lines correspond to the C–H bond distributions in CH_5 species, with the red line specifically representing C–H bonds involved in three-center two-electron bonds.

Calculating the CH_5^+ rate of formation

Here we describe the approach used to compute the frequency at which CH_5^+ is formed, presented in Figure 2c. Instead of focusing on formation (a proton transfer from an oxygen atom to a CH_4), we enumerate CH_5^+ destruction events (a proton transfer from CH_5^+ to an oxygen atom). We record each instance in which a hydrogen in CH_5^+ experiences a nearest neighbor change from carbon to a nearby oxygen. To avoid over-counting events due to fluctuations, we exclude rapid back-and-forth proton transfers by applying an intermittent function checking for the completion of the proton transfer over a 30 fs window. Figure S8 shows the number of excluded proton transfers as a function of the intermittent function time. As observed, convergence is achieved from 30 fs onward.

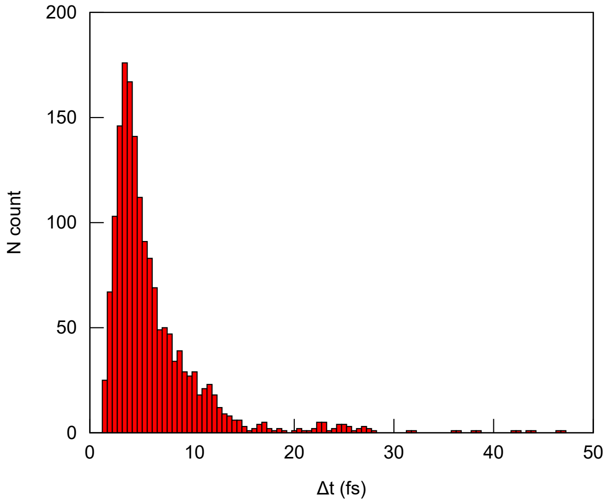

Figure S8. Number of proton transfers from carbon to oxygen that are excluded by the intermittent function.

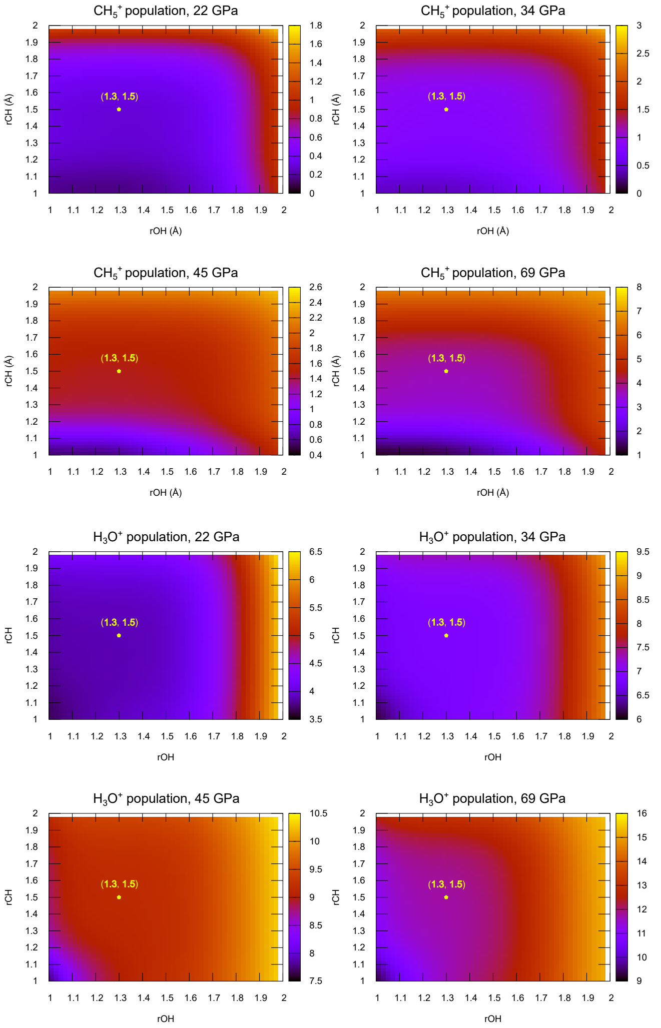

Figure S9. Effect of C–H and O–H cutoff values, namely rCH and rOH, on the CH_5^+ (left half) and H_3O^+ (right half) populations in the 22-69 GPa range at 3000 K. The screening is done for cutoff values ranging from 1 to 2 Å. The (rOH, rCH) point corresponding to the chosen cutoff values is reported in yellow.

9. Vibrational density of states

In Figure S10a, we report the total vibrational density of states (VDOS) for the CH_4/H_2O mixture in the 22–69 GPa range at 3000 K. A blue shift of the bands in the 3000–3200 cm^{-1} region is observed, reminiscent of that reported experimentally via Raman spectroscopy on methane hydrates at increasing pressure (12-45 GPa) [23], albeit at lower temperatures than those studied here.

Additionally, the band around 1200 cm^{-1} shows a change in shape and broadening with increasing pressure, accompanied by an increase in the density of states near 1000 cm^{-1}. A shift of the band around 500 cm^{-1}, associated to hindered rotation and translation is also observed.

In Figure S10b, we show the first vibrational signatures of CH_5^+ in water, after removing contributions from rotational and translational motions, and compare them with the corresponding signal of CH_4, also corrected for rotational and translational contributions. Only CH_5^+ species with lifetime longer than 10 fs has been selected to participate to the average velocity autocorrelation function. Despite the broad peaks due to the strong anharmonicity of CH_5^+, the high temperatures and the possibly the limited statistics (resulting from its short lifetime and the short *ab initio* MD trajectories), two specific features associated with CH_5^+ vibrational modes stand out. Interestingly, the red shift of the C–H stretching in the 2500–3000 cm^{-1} range relative to CH_4 is consistent with the weakening of the C–H bond in CH_5^+, as deduced from the increased bond length reported in the analysis in Figure S7c. The increase in the VDOS around 1000 cm^{-1} in the total VDOS spectra with increasing pressure may reflect a higher concentration of CH_5^+, according to the features observed in its vibrational density of states. The VDOS has been computed within the time-correlation function formalism, from the DFT-MD trajectories as the Fourier transform of the atomic velocity autocorrelation function [24,25]:

$$\mathrm{VDOS}(\omega) = \frac{1}{E_k} \sum_{i=1}^{N} \int_{-\infty}^{\infty} \langle \mathbf{v}_i(0) \cdot \mathbf{v}_i(t) \rangle \, e^{-i\omega t} \, dt, \tag{3}$$

where $v_i(t)$ is the velocity vector of atom i at time t in mass-weighted coordinates and E_k is the averaged kinetic energy of the system. The angular brackets denote a statistical average of the correlation function.

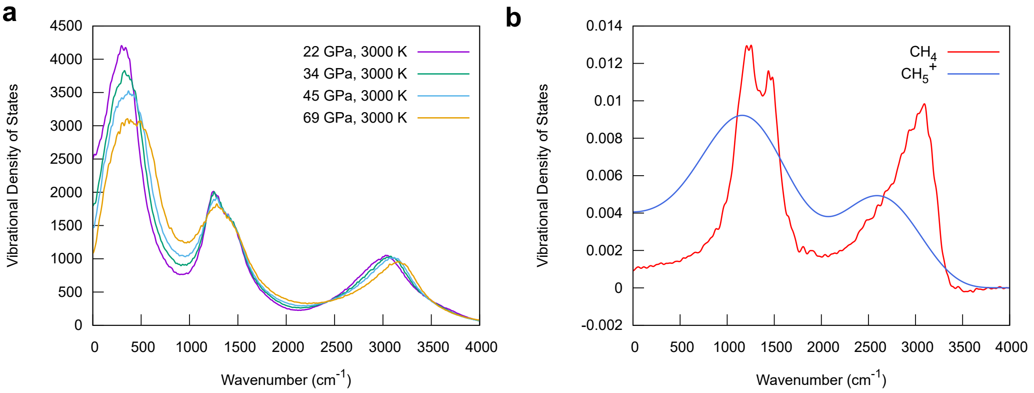

Figure S10. Panel a: Total vibrational density of states (VDOS) for the CH_4/H_2O mixture in the 22–69 GPa pressure range at 3000 K. The yellow, blue, green, and purple curves correspond to 69, 45, 34, and 22 GPa, respectively.**Panel b:** Vibrational signatures of CH_5^+ (blue curve) and CH_4 (red curve) at 45 GPa and 3000K after removing contributions from rotational and translational motions.

10. M3 mechanism and lifetime distributions of ethylene and formaldehyde

The M3 mechanism is an elongation mechanism involving the formation of a double-bond intermediate. The first step proceed trough an elimination reaction that lead to a double-bond intermediate (e.g. ethene, formaldehyde). The rsweaction than continues trough an addition step where the double-bonded intermediate acts as an electrophilic partner to a neighboring molecule (hydrocarbon, alcohol or water). The M3 mechanism can proceed via either a base-catalyzed or an acid-catalyzed pathway. In the acid-catalyzed pathway (shown in Figure 5c of the main text), the double-bonded intermediate is produced from a tricoordinated hydrocarbon or alcohol cation, previously generated via dehydrogenation of a pentacoordinated carbocation (as observed in the M1 mechanism).

By contrast, in the base-catalyzed pathway the double bond intermediate derives from an anionic species generated by deprotonation of a hydrocarbon or an alcohol by OH^-. In Figure S11, we illustrate the spontaneous

formation of 1,1-ethanediol via the M3 base-catalyzed pathway observed in an unbiased MD simulation at 3000 K and 45 GPa. The reaction begins with the deprotonation of the hydroxyl group of ethanol by an OH^- ion (Structures I, Figure S11), releasing a water molecule. The resulting $CH_3CH_2O^-$ anion (Structure II, Figure S11) evolves to form a C=O double bond, yielding an acetaldehyde molecule and releasing a hydride ion (H^-), which subsequently combines with a nearby proton (H^+) to form molecular hydrogen (H_2) (Structure III, Figure S11). Subsequently, a water molecule performs a nucleophilic attack on the acetaldehyde. This condensation step results in the breaking of the C=O bond and the addition of a hydroxyl group, leading to the 1,1-ethanediol molecule (Structures IV, Figure S11). It is important to note that the M3 base-catalzed mechanism was also characterized in metadynamics DFT-MD simulations, where ethane can also be deprotonated from OH^-, forming ethene as an intermediate.
In Figure S12, we present the lifetime distributions of ethene (top) and formaldehyde (bottom), two of the M3 double bonded intermediates observed in both acid and base catalyzed variants, at 3000 K under varying pressure conditions. The distributions clearly demonstrate a decrease in intermediate stability with increasing pressure, as evidenced by the shift toward shorter lifetimes. This behavior highlights the reduced stability of π bonds at elevated pressures, which can be attributed to their relatively large spatial occupancy.

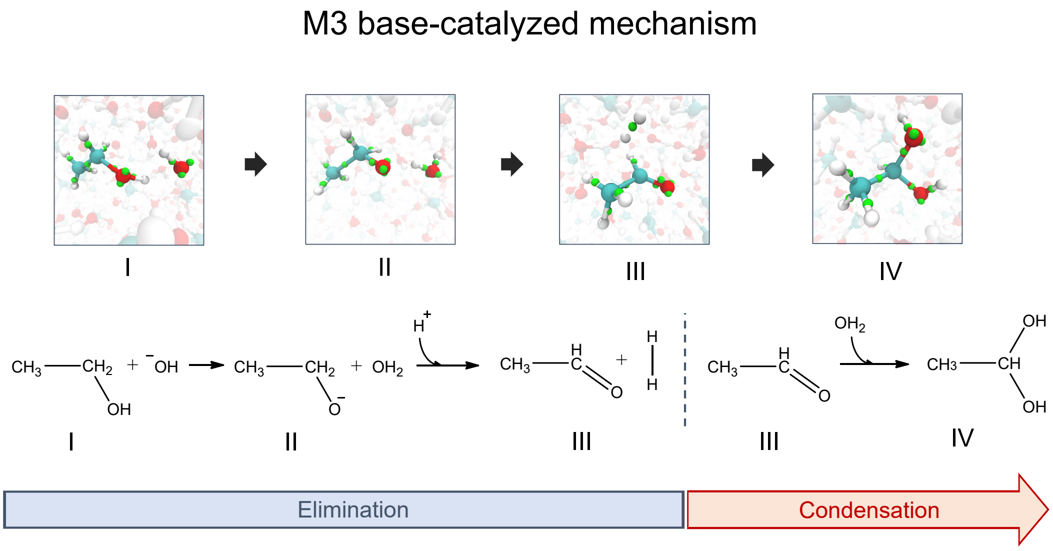

Figure S11. Scheme of the reaction mechanisms, along with the Wannier centers associated with the reactive structures for the M3 base-catalyzed mechanism.

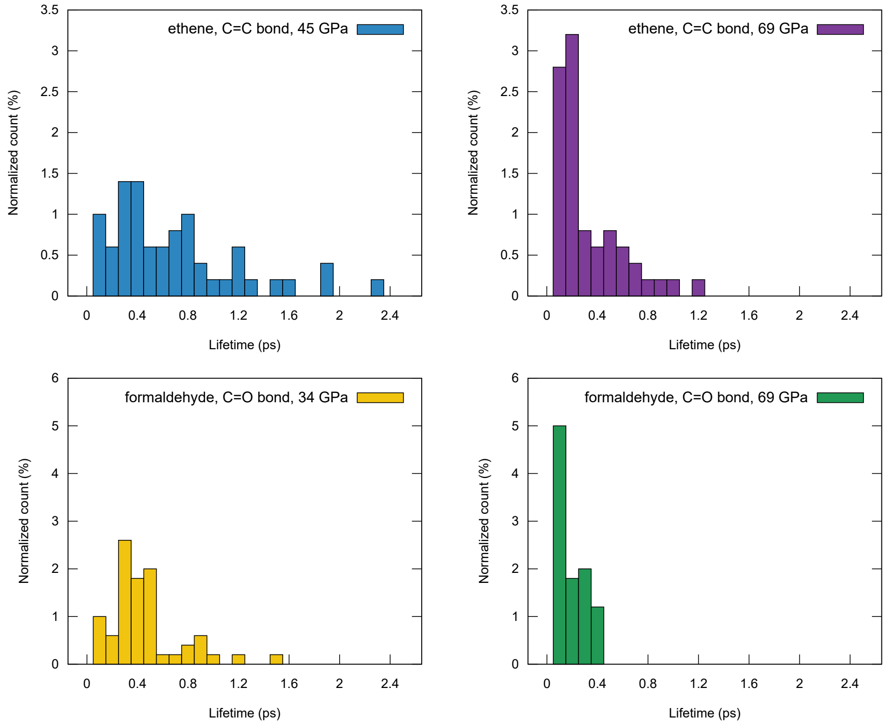

Figure S12. Top Panel: Lifetime distributions of ethene (C_2H_4) at 45 GPa (blue, left) and 69 GPa (purple, right) at 3000 K. **Bottom Panel**: Lifetime distributions of formaldehyde (CH_2O) at 34 GPa (yellow, left) and 69 GPa (green, right) at 3000 K. Each distribution is based on 50 DFT-MD simulations using a 488-atom simulation box, totaling 200 DFT-MD simulations across all pressures.

11. Formation of quaternary hydrocarbon structure along unbiased DFT-MD

In Figure S13, we follow the transformation from a ternary to a quaternary hydrocarbons species, by computing the coordination numbers of the reactive atoms along the unbiased DFT-MD simulation at 45 GPa and 3000 K. In particular, the coordination numbers of the hydrocarbon central carbon with respect to all the carbons (black curve) and oxygen atoms of the system (red curve) are reported as a function of time.

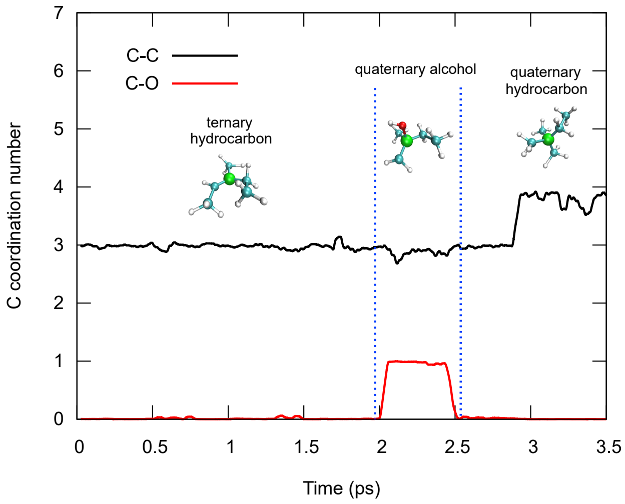

Figure S13. Evolution of the coordination number of the hydrocarbon central carbon (in green) during its spontaneous transformation from a ternary to a quaternary species in an unbiased MD simulation. The red line depicts the C–O coordination number, while the black line shows the C–C coordination number.

12. SPRINT metadynamics

The SPRINT coordinates[26,27] are defined as:

$$S_i = \sqrt{N} \times \lambda^{\max} \times v_i^{\max,\text{sorted}}, \quad i = 1, 2, ..., N \tag{4}$$

where N is the number of atoms, $\lambda^{\max}$ and $v^{\max,\text{sorted}}$ are the largest eigenvalue and corresponding eigenvector (with sorted entries) of the smooth adjacency matrix of atoms, composed by a_{ij} elements:

$$a_{ij} = \frac{1 - (r_{ij}/r_0)^n}{1 - (r_{ij}/r_0)^m} \tag{5}$$

where r_{ij} are interatomic distances and r_0, n, and m are parameters depending on the typical bond lengths of the system under consideration. The parameters for the set of switching functions, specifically the r_0, n, and m parameters, are provided for all atom pairs in Table S4.

Table S4

Atoms	R_0 (Å)	N	M
C-C	2.40	4	16
C-H	1.10	8	16
C-O	2.70	8	12
H-H	0.74	8	16
H-O	0.95	8	16
O-O	1.40	8	16

The S_i SPRINT variables, obtained by diagonalizing the smooth interatomic adjacency matrix, are centered on each atom of the system and capture changes in both its short- and long-range connectivity. We have benefited from the dimensional reduction allowed by the magnitude-ordering, which introduces correlations among the values of the S_i. For this reason, in between 16 and 24 out of the SPRINT variables were biased in our metadynamics simulations. Table S5 provides details of the SPRINT simulations performed on a $CH_{4/}H_2O$ box containing 732 atoms, which led to the formation of a diamond-like structure (see reaction mechanism in Figure 4b of the main text). The table lists the simulation lengths, the maximum value of the metadynamics bias, the metadynamics parameters, the number of biased SPRINT variables (N_{SPRINT}) and the heaviest hydrocarbon species formed during the metadynamics. Similarly, Table S6 summarizes the same parameters for six independent SPRINT simulations from box containing 488 atoms, which resulted in the formation of complex hydrocarbon species (see reaction mechanism for 3-methyl pentane formation in Figure S14). For all the metadynamics the Hills deposition time is of 100 steps. The *ab initio* MD simulations, have been coupled with the metadynamics algorithm[28–31] via the Plumed plugin[32].

Table S5. Parameters for the SPRINT metadynamics simulations leading to the formation of a quaternary diamond-like structure from a simulation box containing 722 atoms, consisting of one 3-methylpentane molecule in a $CH_{4/}H_2O$ mixture at 3000 K and 45 GPa. The first column reports the simulation time, while columns two through six provide details on the metadynamics hill height, hill width, the number of biased SPRINT coordinates, and the heaviest hydrocarbon species formed.

Time (ps)	Maxium bias (k_BT)	Hills height (k_BT)	Hills width	N	Hydrocarbon
7.8	16	1.4	2.5	24	3,3-dimethylhexane

Table S6. Parameters for the SPRINT metadynamics simulations leading hydrocarbon elongation and branching from $CH_{4/}H_2O$ systems containing 488 atoms at 3000 K and 45 GPa. The first column lists the simulation time. Columns two through six detail the metadynamics hill height, hill width, the number of biased SPRINT coordinates, and the heaviest hydrocarbon species formed.

Time (ps)	Maxium bias (k_BT)	Hills height (k_BT)	Hills width	N	Hydrocarbon
9	27.7	1.4	2.5	16	3-methylpentane
17	27.8	1.4	1.5	22	2-methylpentane
17	21.9	1.4	2.5	24	pentane
16	17.2	0.4	2.5	24	propane
15	29.0	1	2.5	24	propane
14	23.3	1.4	2.5	24	propane

13. Formation of 3-methylpentane from metadynamics

In Figure S14, we report the complete reaction pathway for the formation of 3-methylpentane from ethane and methanol, observed at 45 GPa and 3000 K during a SPRINT metadynamics simulation. As can be seen, the formation process involves only the two acid-catalyzed M1 and M2 mechanisms and the base-catalyzed M3 mechanism, previously characterized in unbiased simulations and reported in Figure 5 of the main text and Figure S11 of the Supporting Information, respectively.

The reaction begins with an ethane molecule condensing with a CH_3^+ cation via an M1 mechanism, leading to the formation of propane. The resulting propane molecule then undergoes C–H protolytic cleavage, producing an undercoordinated $(CH_3)_2CH^+$ carbocation and releasing H_2. This carbocation subsequently relaxes via an M3 mechanism, yielding the double-bond intermediate propylene (CH_3CHCH_2).

The propylene then condenses with a formaldehyde molecule (previously generated via the M3 base-catalyzed mechanism), producing 2-methylpropan-1-ol. This alcohol is converted into 2-methylbutane through an M2 mechanism: the hydroxyl group is protonated and leaves as H_2O, forming a trivalent carbocation ($(CH_3)_2CHCH_2^+$) that subsequently condenses with a methane molecule to yield 2-methylbutane. Chain growth continues via an M1 mechanism, in which a CH_3^+ cation react with the end of the chain, ultimately producing the 3-methylpentane molecule.

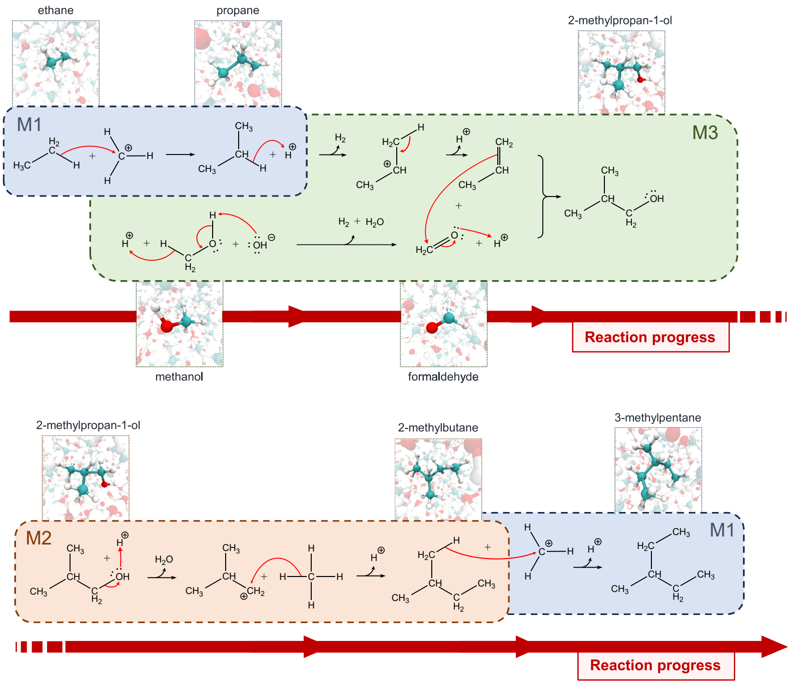

Figure S14. Scheme of the reaction mechanism for 3-methylpentane formation, containing a ternary hydrocarbon structure, at 3000K and 45 GPa obtained by SPRINT-based metadynamics simulation.The superacid M1, M2 and a base-catalyzed M3 mechanisms, previously characterized by unbiased DFT-MD simulations, are identified.

14. DFT: labeling for MLIPs

We selected configurations sampled from DFT-MD and MLIP-MD to perform accurate single-point calculations. These datasets were then used to train MLIPs at different levels of accuracy - this therefore constitutes a "labeling" step. For all these calculations, we increased the SCF convergence cutoff to 10^{-7} Ha, and switched to TZVP-MOLOPT basis sets. We first performed calculations at the PBE level, which constitutes the "PBE large basis" dataset used later for training ("PBE small basis" being the DFT-MD reference). Then, we performed calculations at the hybrid functional level of theory, using PBE0[33]. We have used the FIT3 auxiliary basis set and truncated the Coulomb potential at half the system cell (6.4 Å), following guidelines to accelerate such calculations[34]. CP2K input scripts for both PBE and PBE0 are provided as Supplemental Material.

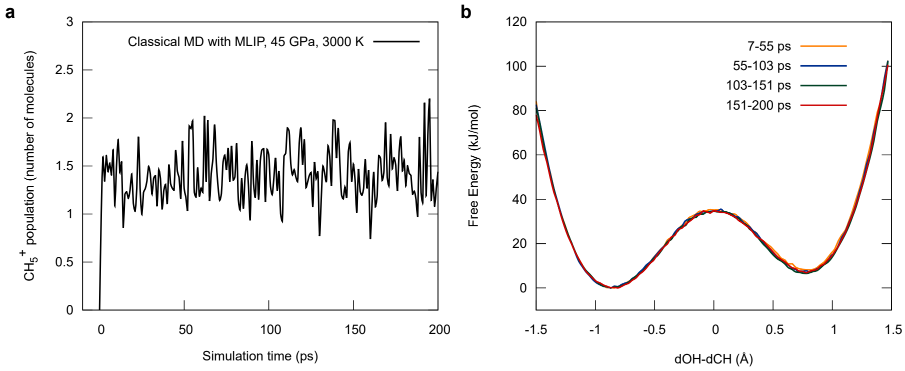

Figure S15. Panel a: The black curve shows the population of CH_5^+, averaged over 1 ps intervals, as a function of simulation time at 3000 K and 45 GPa from MLIP-MD simulations. **Panel b:** Evolution of the free energy profiles of CH_5^+ formation from H_3O^+ + CH_4 during the 200 ps trajectory.

15. CH_5^+ populations and free energy of formation from MLIP-MD

In Figure S15a, we show the time evolution of the CH_5^+ population obtained from MLIP-MD simulations over 200 ps at 45 GPa and 3000 K. After a brief equilibration phase, the CH_5^+ population remains stable throughout the simulation.

Such stability is consistent with the associated free energy of CH_5^+ formation reported in Figure S15b. From 7 to 200 ps, the free energy profile does not exhibit any significant change when sampled each quarter of this portion of the trajectory. Details on the MLIP training and generation can be found in Section 16 of this document.

16. Machine learning interatomic potentials

MLIPs: training

We exclusively used the Allegro architecture[35], a strictly-local, equivariant neural network. We implemented symmetries of the SO(3) group on geometric inputs and internal features. We used a radial cutoff of 4 Å, 64 features for irreps, 2 tensor product layers, and a maximal rotation order l for spherical harmonics set to 2. Input scripts describing the selected architecture and the optimization process are included as Supplemental Material.

We followed an iterative training strategy in which successive MLIPs are used to generate new configurations using molecular dynamics, which are then labeled using DFT. First, we sampled configurations from DFT-MD calculations at 3000 K and 45 GPa, by downsampling a 24 ps long trajectory (equilibration being excluded) using a stride of 50 fs. This amounts to 480 configurations, out of which 50 are used for validation. In a first iteration, after training a first model on the DFT dataset, we performed a single molecular dynamics simulations of 700 ps, from which we sampled 70 frames (one every 10 ps). We then labeled these configurations, added them to the original DFT dataset, and trained a new model. We repeated this operation 4 times, although we performed each time 8 independent molecular dynamics simulations of 150 ps, sampling new structures every 5 ps. In the end, this leads to a dataset of $480 + 70 + 240 \times 4 = 1510$ structures, out of which 50 are used for validation.

Training is performed using a single MI250 GPU, with the Adam optimizer and default hyperparameters. The loss function includes both the mean squared error of the per-atom energies, and the mean squared error of the atomic forces. We adopt an initial learning rate of 10^{-5}, and schedule its reduction by a factor of 0.35 following a plateau during 50 epochs of the loss. We select the model that minimizes the validation loss during training. On the final datasets, we obtain validation set root mean squared errors on the energies and the forces on the order of 6 meV/atom and 175 meV/Å (PBE), and 7 meV/atom and 204 meV/Å (PBE0). The errors on the atomic forces are relatively large compared to typical figures obtained for simpler chemical systems at atmospheric conditions. This is associated to the highly reactive environment at play; the figures obtained compare favorably to the ones reported previously for pure hydrocarbon mixtures under extreme conditions[36]. We have also prepared a challenging test set, composed of configurations spanning the chemical diversity observed in our molecular dynamics simulations. First, we have projected our datasets onto a 204-dimensional space of the concentrations, in carbon and oxygen, of 102 identified chemical species. Then, using farthest point sampling, we have selected 1000 configurations corresponding to the ones showing the highest chemical diversity. We report here the training, validation, and test metrics obtained for all three models used (Tables S7 and S8). In addition, we present error distributions on the atomic forces as a function of the norm of the target forces (Figure S16), an example of optimization result for the "PBE - small basis" model (Figure S17), and the convergence of free energy profiles

with respect to the number of beads used in our normal-mode path integral molecular dynamics simulations (Figure S18).

MLIPs: molecular dynamics

All MLIP-based molecular dynamics simulations have been performed using LAMMPS [37] (7 Feb 2024), patched for Allegro and for D3 dispersion corrections which we implement analytically. We use interaction and coordination number cutoffs of 11.2 Å for D3 Becke-Johnson corrections. To estimate free energy profiles, we perform 8 independent 10 ps long simulations, in the NVT ensemble at 3000 K, using a time step of 0.5 fs, and a Nosé-Hoover chain of three thermostats with a time constant of 50 fs. We increased the MLIP-MD simulation time to 200 ps and found that the free energies were invariant and that the CH5+ population was stable (see Figure S15).

To take into account nuclear quantum effects, we perform normal-mode path integral molecular dynamics with the stochastic path integral Langevin equation (PILE) thermostat [38], using a damping time of 50 fs on the centroid mode. We use 6 polymer beads and have tested for convergence, as reported in Figure S18.

Table S7. Force errors on training, validation and test datasets.

MAE / RMSE (meV/Å)	Train	Validation	Test
PBE - small basis	122 / 171	122 / 181	142 / 215
PBE - large basis	114 / 159	118 / 175	136 / 206
PBE0	125 / 175	132 / 204	156 / 246

Table S8. Per-atom energy errors on training, validation and test datasets.

MAE / RMSE (meV/atom)	Train	Validation	Test
PBE - small basis	11.6 / 14.9	5.0 / 6.3	4.9 / 6.0
PBE - large basis	12.3 / 15.5	4.6 / 6.1	7.4 / 8.8
PBE0	12.4 / 15.3	5.9 / 7.4	7.9 / 9.8

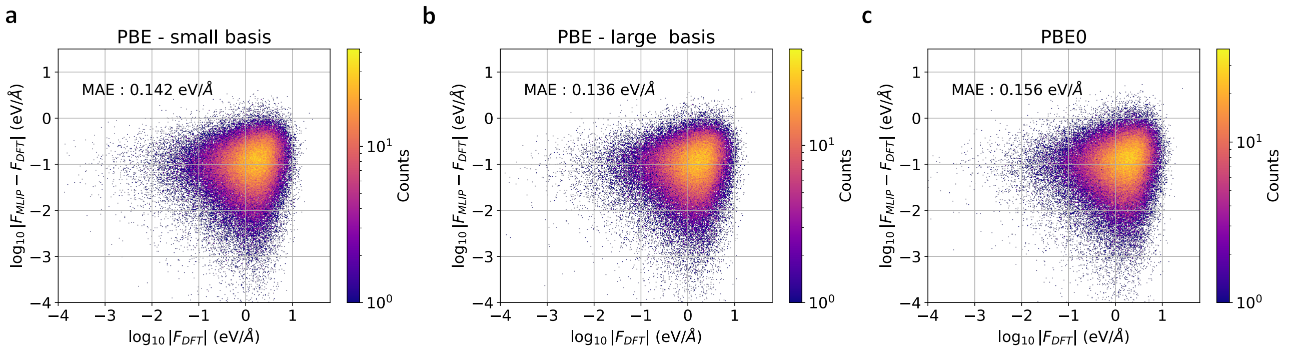

Figure S16. Test set atomic force error distributions as a function of the reference force norms for the three MLIPs implemented (**a**: "PBE - small basis", **b**: "PBE - large basis", **c**: "PBE0") in logarithmic scale.

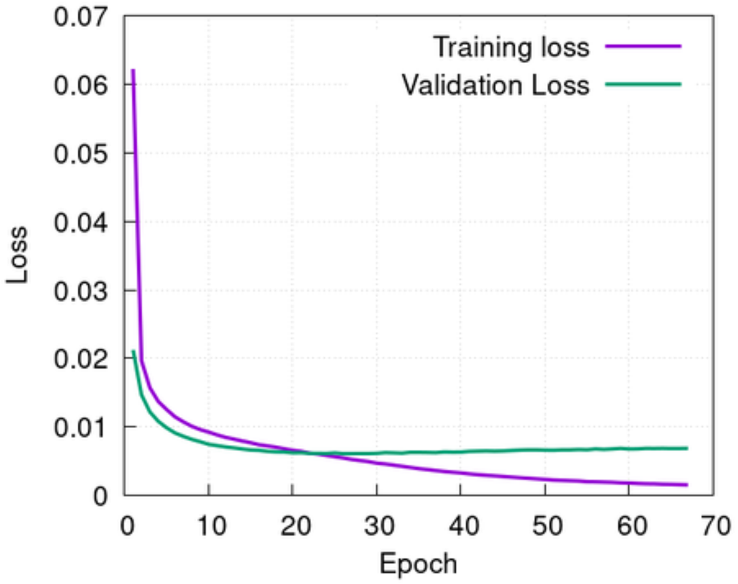

Figure S17. Evolution of the training and validation loss during optimization for the "PBE - small basis" dataset.

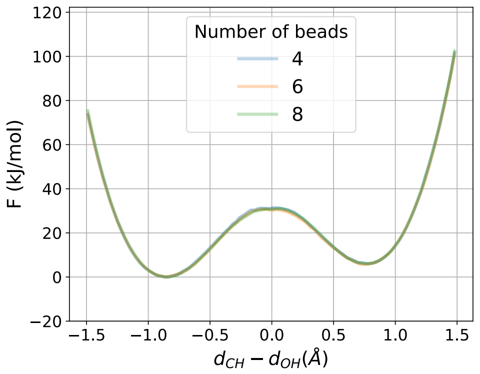

Figure S18. Free energy profile associated with the proton transfer from H_3O^+ to CH_4 obtained from normal-mode path integral molecular dynamics simulations, with either 4, 6, or 8 polymer beads.

References

[1] L. Martínez, R. Andrade, E. G. Birgin, J. M. Martínez, J. Comput. Chem. **2009**, 30, 2157.
[2] J. VandeVondele, M. Krack, F. Mohamed, M. Parrinello, T. Chassaing, J. Hutter, Comput. Phys. Commun. **2005**, 167, 103.
[3] J. P. Perdew, K. Burke, M. Ernzerhof, Phys. Rev. Lett. **1996**, 77, 3865.
[4] S. Grimme, S. Ehrlich, L. Goerigk, J. Comput. Chem. **2011**, 32, 1456.
[5] M.-S. Lee, S. Scandolo, Nat. Commun. **2011**, 2, 185.
[6] V. Rozsa, D. Pan, F. Giberti, G. Galli, Proc. Natl. Acad. Sci. U.S.A. **2018**, 115, 6952.
[7] G. A. Olah, Carbocation Chem. **2004**, pages 7–41.
[8] G. A. Olah, G. Klopman, R. H. Schlosberg, J. Am. Chem. Soc. **1969**, 91, 3261.
[9] G. A. Olah, G. S. Prakash, J. Sommer, Science **1979**, 206, 13.
[10] D. Kim, M. L. Klein, J. Phys. Chem. B **2000**, 104, 10074.
[11] S. Raugei, M. L. Klein, J. Phys. Chem. B **2002**, 106, 11596.
[12] B. Michelet, A. Martin-Mingot, J. Rodriguez, S. Thibaudeau, D. Bonne, Chem. Eur. J. **2023**, 29, e202300440.
[13] P. Bourbon, K. Vitse, A. Martin-Mingot, H. Geindre, F. Guégan, B. Michelet, S. Thibaudeau, Nat. Commun. **2024**, 15, 7435.
[14] D. Marx, M. Parrinello, Nature **1995**, 375, 216.
[15] S. D. Ivanov, O. Asvany, A. Witt, E. Hugo, G. Mathias, B. Redlich, D. Marx, S. Schlemmer, Nat. Chem. **2010**, 2, 298.
[16] X. Huang, L. M. Johnson, J. M. Bowman, A. B. McCoy, J. Am. Chem. Soc. **2006**, 128, 3478.
[17] P. Kumar, D. Marx, Phys. Chem. Chem. Phys. **2006**, 8, 573.
[18] X. Huang, A. B. McCoy, J. M. Bowman, L. M. Johnson, C. Savage, F. Dong, D. J. Nesbitt, Science **2006**, 311, 60.
[19] A. Brown, A. B. McCoy, B. J. Braams, Z. Jin, J. M. Bowman, J. Chem. Phys. **2004**, 121, 4105.
[20] S. X. Tian, J. Yang, J. Phys. Chem. A **2007**, 111, 415.
[21] D. Marx, M. Parrinello, Science **1999**, 284, 59.
[22] P. R. Schreiner, Angew. Chem. Int. Ed. **2000**, 39, 3239.
[23] S. Schaack, U. Ranieri, P. Depondt, R. Gaal, W. F. Kuhs, A. Falenty, P. Gillet, F. Finocchi, L. E. Bove, The Journal of Physical Chemistry C **2018**, 122, 11159.
[24] D. R. Galimberti, S. Bougueroua, J. Mahé, M. Tommasini, A. M. Rijs, M.-P. Gaigeot, Faraday Discussions **2019**, 217, 67.
[25] M.-P. Gaigeot, Physical Chemistry Chemical Physics **2010**, 12, 3336.
[26] F. Pietrucci, W. Andreoni, Phys. Rev. Lett. **2011**, 107, 085504.
[27] F. Pietrucci, W. Andreoni, J. Chem. Theory Comput. **2014**, 10, 913.
[28] A. Laio, M. Parrinello, Proc. Natl. Acad. Sci. U.S.A. **2002**, 99, 12562.
[29] G. Bussi, A. Laio, Nat. Rev. Phys. **2020**, 2, 200.
[30] F. S. Brigiano, M. Gierada, F. Tielens, F. Pietrucci, ACS Catal. **2022**, 12, 2821.
[31] X. Li, F. S. Brigiano, S. Pezzotti, X. Liu, W. Chen, H. Chen, Y. Li, H. Li, X. Lin, W. Zheng, et al., Nat. Chem. **2024**, pages 1–6.
[32] G. A. Tribello, M. Bonomi, D. Branduardi, C. Camilloni, G. Bussi, Comput. Phys. Commun. **2014**, 185, 604.
[33] C. Adamo, V. Barone, J. Chem. Phys. **1999**, 110, 6158.
[34] M. Guidon, J. Hutter, J. VandeVondele, J. Chem. Theory Comput. **2010**, 6, 2348.
[35] A. Musaelian, S. Batzner, A. Johansson, L. Sun, C. J. Owen, M. Kornbluth, B. Kozinsky, Nat. Commun. **2023**, 14, 579.
[36] B. Cheng, S. Hamel, M. Bethkenhagen, Nat. Commun. **2023**, 14, 1104.
[37] A. P. Thompson, H. M. Aktulga, R. Berger, D. S. Bolintineanu, W. M. Brown, P. S. Crozier, P. J. In't Veld, A. Kohlmeyer, S. G. Moore, T. D. Nguyen, et al., Comput. Phys. Commun. **2022**, 271, 108171.
[38] M. Ceriotti, M. Parrinello, T. E. Markland, D. E. Manolopoulos, J. Chem. Phys. **2010**, 133.
